# A herbaceous species provides insights into drought-driven plant adaptation

**DOI:** 10.1093/jxb/erac485

**Published:** 2023-02-04

**Authors:** Kate M Johnson, Leila R Fletcher

**Affiliations:** School of the Environment, Yale University, New Haven, CT 06520, USA; Department of Organismic and Evolutionary Biology, Harvard University, Cambridge, MA 02138, USA; School of the Environment, Yale University, New Haven, CT 06520, USA

**Keywords:** Adaptation, Arabidopsis, cavitation, drought, evolution, lignification, woodiness

## Abstract

This article comments on:

**Thonglim A, Bortolami G, Delzon S, Larter M, Offringa R, Keurentjes JJB, Smets E, Balazadeh S, Lens F**. 2023. Drought response in Arabidopsis displays synergistic coordination between stems and leaves. Journal of Experimental Botany **74**, 1004–1021

**Pinpointing the mechanisms and traits driving plant drought resistance is a challenging task. Previous research by** **Thonglim *et al.* (2021)** **found that the thickness of the pit membrane, the site of air entry into the water-conducting cells (the xylem) of flowering plants, was the best predictor of resistance to drought-induced cavitation in *Arabidopsis thaliana*. This provides evidence that greater pit membrane thickness is a mechanistic driver of drought resistance in this and possibly other flowering plants (angiosperms). In the paper discussed here,** **Thonglim *et al.* (2023)** **find that not only pit membrane thickness, but also the overall degree of lignification (thickening of secondary plant cell walls by deposition of the complex polymer lignin) in the main stem, associated with a range of leaf and stem traits, is correlated with greater drought resistance in *A. thaliana*.**

## Insights from Arabidopsis

*Arabidopsis thaliana* (henceforth Arabidopsis), beloved thale-cress, has been instrumental in developing our understanding of plant function. With its expansive natural range, the abundance of existing mutants, and the availability of information about its genetic profile, Arabidopsis also presents the ideal system to study the intrinsic physiological drivers of drought resistance (1001 Genomes Consortium, 2016; Weigel and Mott, 2009). Utilizing this system, Thonglim *et al.* (2023) begin to tackle a major challenge in plant science—the integration of plant physiology, growth, and genetics.

Thonglim *et al.* highlight that different modes of drought adaptation can be present within a single species. This raises the question of what it means to ‘tolerate’ drought, an interesting concept explored recently in relation to whole-plant growth in Arabidopsis (Fletcher *et al.*, 2022). Some naturally occurring genotypes (ecotypes) of this annual plant have been found to complete their life cycles rapidly, while other ecotypes are longer lived (Mitchell-Olds and Schmitt, 2006; Fletcher *et al.*, 2022). Thonglim *et al.* suggest that stem lignification may be higher in longer lived stems, which would be consistent with the idea that, as longer lived genotypes are more likely to experience stress, they may have greater need to invest in strategies for drought resistance. Higher lignification was also found to be coupled with reduced stomatal conductance, leading to lower water loss and therefore greater capacity to survive drought (Thonglim *et al.*, 2023). In contrast, the lack of lignification, presence of thinner intervessel pit membranes, and higher stomatal conductance in more drought-sensitive genotypes (Thonglim *et al.*, 2023) together imply that these may have higher photosynthetic rates to allow for rapid growth and quick life cycle completion in non-stressful periods.

## Adaptation across scales

**Fig. 1.**
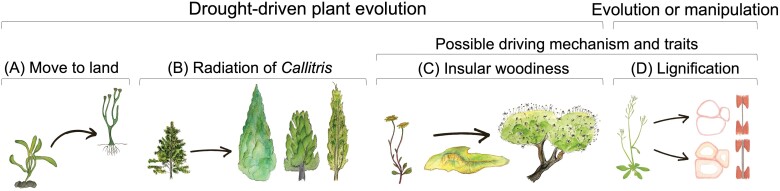
Drought plays a major role in plant adaptation. Examples of this include (from left to right): (A) the move of plants from water to land (B) the radiation of the genus *Callitris* into arid climates, and (C) the transition from herbaceousness to woodiness on islands. Thonglim *et al.* (2023) found that increased lignification was correlated with increased xylem pit membrane thickness in Arabidopsis (D), leading to greater drought resistance via more resistant P50s along with coordination of a number of stem and leaf traits. Increased lignification and pit membrane thickness (D) may offer a possible mechanistic explanation, not only for drought resistance in Arabidopsis (Thonglim *et al.*, 2023), but also for drought resistance in insular woody species (C). Finding that increased lignification confers drought resistance in both mutants and naturally occurring ecotypes indicates that lignification can be the result of selective pressure or human manipulation of the Arabidopsis genome (D). This information not only provides insights into how drought impacts plant adaptation, but will also be essential for identifying and selecting for drought resistance, which is imperative as drought becomes an increasing threat to plant survival.

As well as revealing possible life history-driven differences in drought strategies within Arabidopsis, by utilizing both ecotypes and mutants, the results presented by Thonglim *et al*. can be interpreted in the context of drought-driven plant evolution at different scales. Mutants show how small, human-manipulated, changes to a genome can influence drought resistance and tolerance, and ecotypes reveal how natural pressures have shaped this species’ drought strategies. The key findings from both can be considered in the context of how drought resistance has shaped and continues to shape the evolution of the plant water transport system (Fig. 1).

## Drought as a major driver of plant evolution

In moving from water to land, plants faced a significant, novel challenge—how to cope with water limitation. Building water and sugar transport systems for terrestrial environments required a suite of adaptations beginning ~475 million years ago. Tolerance to desiccation is thought to have been a key early step, with a desiccation tolerance pathway recently found in an algal ancestor of modern plants (Zhao *et al.*, 2019). Stomata and the waxy cuticle evolved early to reduce dehydration. Water- and nutrient-conducting cells emerged, joined through abundant plasmodesmata, followed by the evolution of secondary cell walls, lignified tracheids, and vessel elements, providing support for water to move under tension (Lucas *et al.*, 2013). In addition, specialized pits evolved to form connections for unhindered water transport between tracheids and vessels (Lucas *et al.*, 2013).

Coping with water deficit was essential to colonizing land (Fig. 1A), and recent research has shown that drought may have also been a driver of the diversification of stelar xylem arrangements in early vascular plants (Bouda *et al.*, 2022). By modelling air embolism spread, Bouda *et al.* (2022) suggest that rapid divergence from the early ‘cylindrical’ stele into more complex shapes allowed plants to better avoid embolism, by reducing the pathways for embolism to spread through the water transport system. Increasing complexity in xylem network arrangements therefore may have reduced the impact of drought-induced embolism, conferring increased drought resistance.

At a broader scale, aridity has also been shown to be a strong driver of species diversification (Fig. 1B). The radiation of the Australian genus *Callitris*, the most drought-resistant clade of trees, was found to be driven by water availability, with this clade’s wide range of P50s (the level of drought stress where 50% of the water transport system is rendered non-functional due to cavitation) found to be strongly correlated with mean annual precipitation (Larter *et al.*, 2017). It is therefore clear that, at least in some cases, water deficit continues to act as a strong driver of plant vascular evolution.

## Lignification and insular woodiness

The transition from herbaceousness to woodiness on islands is a well-known evolutionary pattern which exemplifies an association between increased lignification and drought resistance. This phenomenon (insular woodiness) is a common and characteristic feature of island floras (Lens *et al.*, 2013). A recent analysis of >1000 insular woody species from across the world revealed that traits related to drought were some of the strongest correlates with the evolution of insular woodiness (Zizka *et al.*, 2022). This is supported by evidence that insular woody species often grow in dry habitats (Lens *et al.*, 2013). Additionally, in some cases, insular woody lineages arose jointly with the appearance of drought in the paleoclimatic record (Hooft van Huysduynen *et al.*, 2021), and some research has shown that stems of insular woody species are more resistant to drought-induced failure of the water transport system than those of their herbaceous relatives (Dória *et al.*, 2018).

Thonglim *et al.* (2023) show that stem lignification (associated with more resistant P50s, and coordination of stem and leaf traits) confers drought resistance, which may offer a mechanistic explanation for how the transition from herbaceousness to woodiness on islands facilitates adaptation to drought (Fig. 1C, D). This is also supported by evidence of high pit membrane thickness in some insular woody *Asteraceae*, which is not present in closely related herbaceous species (Dória *et al.*, 2018). Investigating whether insular woody species are in fact broadly characterized by traits similar to those in the highly lignified Arabidopsis mutant *soc1ful*, and whether these are absent in their herbaceous relatives, would help to reveal whether the suite of drought resistance traits found by Thonglim *et al.* (2023) may drive increased drought resistance in insular woody species. It should be noted, however, that woody species are not inherently more drought resistant than herbaceous species and that Thonglim *et al.* (2023) do not show a complete transition from herbaceousness to woodiness. Nevertheless, the association between increasing aridity and insular woodiness (Lens *et al.*, 2013; Hooft van Huysduynen *et al.*, 2021; Zizka *et al.*, 2022) and between increasing lignification and drought resistance across a spectrum of lignification in herbaceous Arabidopsis (Thonglim *et al.*, 2023) are striking patterns when considered in tandem.

## Understanding what confers drought resistance: a way forward

Identifying the mechanistic traits driving drought resistance, possibly including higher lignification in stems of herbaceous angiosperms, is important for agriculture, forestry, and conservation, especially given the rapid rate of climate warming and increasing frequency and severity of drought. The work by Thonglim *et al*. finds traits associated with known genetic profiles which are mechanistically linked to drought resistance, paving the way for the identification and development of more drought-resistant genotypes, particularly in the many herbaceous crops which underpin global food supply.

With recent research finding evidence of a mechanistic link between drought-induced cavitation (the rapid entry of air into xylem conduits) and tissue damage in both herbaceous (Brodribb *et al.*, 2021) and woody species (Johnson *et al.*, 2021; Mantova *et al.*, 2022), it is clear that elucidating the drivers of xylem cavitation is necessary to understand the control of plant drought resistance. Thonglim *et al.* (2023) show that lignification, pit membrane thickness, and P50 are better predictors of drought resistance than traits such as the stomatal safety margin (SSM; the difference between the water potential at 90% stomatal closure and the water potential associated with stem P50), which supports the idea that pinpointing what drives resistance to cavitation is critically important. With a known mechanistic relationship between cavitation and tissue damage and death (Brodribb *et al.*, 2021; Mantova *et al.*, 2022), we must move beyond finding associations between traits and focus on what drives the disconnection of plant water supply to understand drought resistance.

While understanding the mechanisms behind drought resistance is undoubtedly critical, as the authors highlight, an integrative understanding of drought tolerance and resistance that incorporates plant strategies, genetics, anatomy, and physiology is also key. It is additionally essential to consider all plant organs and the surrounding environment as a continuum (the soil–plant–atmosphere continuum; Sperry, 2003), as highlighted by Thonglim *et al.* through integration of information from both leaves and stems. The below-ground component is another fundamental and understudied part of this continuum, with both woody and herbaceous species found to be sensitive to drought-driven declines in root hydraulic conductance (*K*_r_; Bourbia *et al.*, 2021), and *K*_r_ found to drive stomatal closure in olive (Rodriguez-Dominguez and Brodribb, 2020). Air temperature and water content are also crucial during drought, with recent research finding that high temperature and high vapour pressure deficit can induce drought damage even in the absence of soil drought (Schönbeck *et al.*, 2022). Interdisciplinary research, such as the study by Thonglim *et al.* (2023), and an integrative, holistic perspective across disciplines are both vital in our efforts to characterize and accurately interpret the complex nature of drought tolerance and resistance across plant species.
